# Drug metabolism-related gene ABCA1 augments temozolomide chemoresistance and immune infiltration abundance of M2 macrophages in glioma

**DOI:** 10.1186/s40001-023-01370-6

**Published:** 2023-09-25

**Authors:** Yuanliang Yan, Yuanhong Liu, Qiuju Liang, Zhijie Xu

**Affiliations:** 1 Department of Pharmacy, Xiangya Hospital, Central South University, Changsha, 410008 Hunan China; 2 Department of Pathology, Xiangya Hospital, Central South University, Changsha, 410008 Hunan China; 3 National Clinical Research Center for Geriatric Disorders, Xiangya Hospital, Central South University, Changsha, 410008 Hunan China

**Keywords:** Glioma, ABCA1, Temozolomide, Chemosensitivity, Drug efflux transporter

## Abstract

Gliomas are the most prevalent primary tumor in the central nervous system, with an abysmal 5-year survival rate and alarming mortality. The current standard management of glioma is maximum resection of tumors followed by postoperative chemotherapy with temozolomide (TMZ) or radiotherapy. Low chemosensitivity of TMZ in glioma treatment eventuates limited therapeutic efficacy or treatment failure. Hence, overcoming the resistance of glioma to TMZ is a pressing question. Our research centered on identifying the drug metabolism-related genes potentially involved in TMZ-treated resistance of glioma through several bioinformatics datasets and cell experiments. One efflux transporter, ATP-binding cassette transporter subfamily A1 (ABCA1), was discovered with an upregulated expression level and signaled poor clinical outcomes for glioma patients. The transcript level of ABCA1 significantly elevated across the TMZ-resistant glioma cells in contrast with non-resistant cells. Over-expressed ABCA1 restrained the drug activity of TMZ, and ABCA1 knockdown improved the treatment efficacy. Meanwhile, the results of molecular docking between ABCA1 protein and TMZ showed a high binding affinity. Additionally, co-expression and immunological analysis revealed that ABCA1 facilitates the immune infiltration of M2 macrophages in glioma, thereby stimulating tumor growth and aggravating the poor survival of patients. Altogether, we discovered that the ABCA1 transporter was involved in TMZ chemoresistance and the immune infiltration of M2 macrophages in glioma. Treatment with TMZ after ABCA1 knockdown enhances the chemosensitivity, suggesting that inhibition of ABCA1 may be a potential strategy for improving the therapeutic efficacy of gliomas.

**Supplementary Information:**

The online version contains supplementary material available at 10.1186/s40001-023-01370-6.

## Introduction

Glioma, deriving from glial progenitor or neuroglial stem cells, is comparatively rare in all human tumors. Noticeably, it still accounts for the most prevalent type of primary intracranial tumors and causes alarming mortality with a low 5-year relative survival rate of approximately 35.7% [1, 2]. Gliomas are categorized based on their histological features into astrocytoma, oligodendroglioma, oligoastrocytomas, ependymomas, and glioblastoma multiforme (GBM) [3]. Classification methods of low-grade glioma (LGG) and high-grade glioma (HGG) are most commonly used in clinical practice according to the grade malignancy of gliomas. LGG with a lower malignancy includes astrocytoma (WHO grade I) and oligodendroglioma (WHO grade II), whereas HGG presents deadly malignant behavior such as anaplastic astrocytoma (WHO grade III) and glioblastoma (WHO grade IV) [4]. The current standard regimen for glioma is the maximum safe resection of tumors, followed by postoperative radiotherapy or (and) chemotherapy with temozolomide (TMZ) based on types, grades, molecular characteristics, and clinical symptoms of gliomas [5]. While radiotherapy and chemotherapy after surgical resection improved the quality of life and facilitated the survival benefit of patients, majorities of patients confronted limited effects and even therapeutic failure due to tumor resistance to TMZ [6, 7]. Consequently, exploring the potential resistant mechanisms of TMZ in glioma treatment is crucial to enhance drug chemosensitivity and achieving better efficacy.

Current researches ascribe the activation of the DNA repair system, DNA mismatch repair, drug efflux proteins, glioma stem-like cells, regulation of miRNA, and cytoprotective autophagy to the poor chemosensitivity of TMZ against glioma [8]. Drug efflux proteins are mostly the ATP-binding cassette (ABC) transporters involved in drug distribution and metabolism, influencing the effective drug concentrations at the targeted site and contributing to drug resistance [9, 10]. ABCA1 is a member of the ABC transporters, commonly regarded as mediating the efflux of intracellular cholesterol and lipids to support the biosynthesis of high-density lipoprotein [11, 12]. Interestingly, some anticancer agents have also been reported to be transport substrates for ABCA1, such as platinum, paclitaxel, nitidine, and curcumin, which indicates that ABCA1 may be associated with TMZ efflux and therapy resistance [13–15]. Multiple studies have documented that ABCA1 facilitates cell proliferation and the advances of tumors. The high expression level of ABCA1 heralds reduced survival in patients with breast, ovarian, and colorectal cancers [16–18]. A raised death rate of glioma stem-like cells was discovered after using ABCA1 antagonists followed by TMZ treatment [19]. Nonetheless, studies are still fewer on ABCA1 expression and TMZ resistance in glioma. The role of ABCA1 in the progression and prognosis of glioma also remains a notable question.

Our research focuses on identifying the drug metabolism-related genes or proteins from glioma datasets with bioinformatics technologies and confirmatory experiments to elucidate the resistant mechanism of TMZ treatment in glioma (Additional file 3: Table S1). Only one upregulated drug metabolism-related gene, ABCA1, was screened out. Higher expression levels of ABCA1 in glioma tissues were discovered than in normal brain tissues and indicated poor prognosis in patients with glioma. Overexpressed ABCA1 reduced the drug activity of TMZ in inhibiting the growth of glioma cells, while ABCA1 knockdown followed by treatment with TMZ remarkably increased the death rate of glioma cells and suppressed the cell clone forming. Moreover, molecular docking results revealed high interactions between the ABCA1 protein and TMZ. Additionally, the results of co-expression and immunological analysis demonstrated that ABCA1 participated in the immune regulation and immune cell infiltrating (especially M2 macrophages) in glioma. In conclusion, we revealed that the ABCA1 transporter restrained the chemosensitivity of TMZ and enhanced immune cell infiltration in glioma, intimating that TMZ combined with ABCA1 antagonists was a potential approach to improving the therapeutic efficacy of glioma.

## Materials and methods

### Identification of drug metabolism-related genes in glioma

We were directed at identifying the drug metabolism-related genes potentially involved in the in vivo process of medications administered for glioma treatment. The Gene Expression Omnibus database (GEO database) [20] is an internationally free public repository that embodies plentiful genomics data and comprehensive clinical information on various diseases. As shown in Table 1, three GEO datasets of glioma, including GSE4290 [21], GSE15824 [22], and GSE2223 [23–25], were extracted under the following conditions: ⑴ GEO datasets: glioma, ⑵ organism: homo sapiens, ⑶ study type: expression profiling by arrays. Then, samples in the above datasets were analyzed with GEO2R to obtain the differentially expressed genes (DEGs) in glioma and normal brain tissues. The screening criteria of the DEGs were defined as |logFC |> 1 and *p* < 0.01. Subsequently, we applied the Omicstudio [26] tool for Venn analysis to screen out one co-differentially expressed gene, ABCA1, which was the overlapping area of drug metabolism-related genes and DEGs in glioma.

**Table 1:** Features of three datasets extracted from the GEO database

GEO dataset	Platform	Sample size		Differentially expressed genes	Co-differentially expressed genes	References
		Cancer	Normal			
GSE4290	GPL570	157	23	1801 up-regulated and 1938 down-regulated genes	20 up-regulated and 23 down-regulated genes	[21]
GSE15824	GPL570	30	5	750 up-regulated and 872 down-regulated genes		[22]
GSE2223	GPL1833	50	4	343 up-regulated and 602 down-regulated genes		[23–25]

### Prognostic and diagnostic analysis

Several online databases and tools were utilized to assess whether ABCA1 had potential diagnostic and prognostic value in glioma. The Xiantao tool (https://www.xiantaozi.com/) is an online platform for data analysis and graph drawing that can access and analyze detailed RNA-sequencing data and clinical information on various diseases from the Cancer Genome Atlas (TCGA) database. Hence, we used the Xiantao tool for prognostic analyses. Meantime, we used the BEST database (https://rookieutopia.com/app_direct/BEST/) and the PanCanSurvPlot [27] tool to confirm intensively. All samples involved in the prognosis analysis were categorized into low-expression and high-expression groups by the median expression of ABCA1. The log-rank significance test was used for the statistical methods to analyze the relationship between ABCA1 expression level and the survival rate of patients, with a 95% confidence interval (CI).

Moreover, receiver operation characteristic (ROC) curves plotted by the Xiantao tool were utilized to predict the diagnostic value and effectiveness of ABCA1 in glioma. A total of 1846 samples, 1152 normal samples from the GTEx project (Genotype-Tissue Expression) and 694 tumor samples from the TCGA platform, were included. The area under the curve (AUC) reflects the predictive power of ABCA1, whose numerical value closer to 1 indicates better diagnostic performance, significantly when exceeding 0.9.

### Analysis of ABCA1 expression in glioma and brain tissues

Considering the differential expression of ABCA1 showing statistical and clinical significance in the prognosis of glioma, we further explored its expression level in tumor and normal tissues via multiple databases and statistical software. The expression data from GEO datasets, including GSE4290, GSE15824, and GSE2223, were analyzed by GraphPad Prism (version 8.0.2). In addition, the UCSC database [28] had integrated abundant gene expression data from cancer research projects and individual labs, through which we downloaded the RNA-sequencing data (TCGA TARGET GTEx: RSEM tpm, *n* = 19,131) of pan-cancer and extracted ABCA1 expression data of 662 glioma samples and 1157 normal brain samples. We then compared the expression level of ABCA1 in the glioma tissues and normal brain tissues.

Subsequently, we successively applied three large-scale web databases for further verification. The GEPIA2 [29], a comprehensive web database for large-scale gene expression analysis, was exploited to verify the mRNA expression difference. The UALCAN database [30], an interactive web resource providing high-quality gene and protein expression analysis of various tumors, was utilized to compare the mRNA and protein expression levels. The HPA database [31], aiming at showing all proteins in the human body at the level of cells and tissues, was used to obtain the Immunohistochemical staining pictures of glioma and normal brain tissues.

### Analysis of TMZ activity in glioma

TMZ is an indispensable drug prescribed for the standard chemotherapy of glioma, but TMZ resistance appears in many cases [32]. Thereby, we explored the correlation between ABCA1 overexpression and the drug activity of TMZ, intending to investigate whether ABCA1 enhanced the chemoresistance of TMZ in glioma treatment.

#### TMZ activity and ABCA1 expression

CellMinerCDB [33], a publicly available web resource, integrates a large amount of molecular and pharmacogenomic data of all major cancer cell lines and analyzes pharmacogenomic data for cancer cell lines. We investigated the drug activity of TMZ and ABCA1 expression levels using CellMinerCDB. The cell line set of GDSC-MGH-Sanger was selected. The y-axis is drug activity (- log10 [IC50M]), and the x-axis is mRNA expression (log2).

#### Cell culture

Glioma cells, including U118, TMZ-resistant U118 (U118-R), T98G, and TMZ-resistant T98G (T98G-R), were established in our previous study [34] and cultured in a cell incubator named Dulbecco’s modified Eagle’s medium (Gibco, United States) adding with 100 U/ml penicillin/streptomycin (Gibco, United States) and 10% fetal bovine serum (Gibco, United States) under the circumstances of 5% CO_2_ at 37 ℃. ABCA1 siRNA was used to knock down specific base sequences of ABCA1, including GCTAGAACCCATAGCAACA (siABCA1-1) and CCAGCCAGCTTTGTCGTAT (siABCA1-2). U118-R and T98G-R cells were respectfully and randomly divided into two groups. One group added phosphate-buffered saline as the control group and another supplement with TMZ as the experimental group.

#### Western blot

The glioma cells were incubated in Lysis buffer (Thermo Scientific, United States) on ice for 30 min. Proteins were obtained from centrifugation of cell lysates under 14,000 rpm for 15 min at 4 ℃. Actin antibody (C-2) (sc-8432, Santa Cruz Biotechnology) was the primary antibody used as an internal control. Afterward, a BCA kit (Thermo Scientific, United States) was exploited to determine the protein concentrations. The following step was separating proteins with SDS-PAGE and transferring them onto the polyvinyl difluoride (PVDF) membranes (Millipore, United States). These PVDF membranes were then sealed with TBST for one hour at room temperature, supplemented with 5% non-fat dry milk, and incubated for 12 h at 4 ℃ with ABCA1 monoclonal antibody (#21676, signalway antibody). We eventually detected the above protein bands via the Bio-Rad GelDoc XR + IMAGELAB system.

#### Real-time PCR

The total mRNA of ABCA1 was extracted using Trizol reagent (Invitrogen, United States) and then switched into cDNA by reverse transcription using the cDNA synthesis kit (Takara, China). Real-time PCR was followed at 95 ℃ for 5 min, 40 amplification cycles under 95 ℃ for 15 s, and 56 ℃ for 30 s. Primers of ABCA1 were 5ʹ- CAGGCTACTACCTGACCTTGGT-3ʹ (forward) and 5ʹ- CTGCTCTGAGAAACACTGTCCTC-3ʹ (reverse). Additionally, β-actin was used for the internal control gene, whose primers were 5ʹ- CACCATTGGCAATGAGCGGTTC-3ʹ (forward) and 5ʹ- AGGTCTTTGCGGATGTCCACGT-3ʹ (reverse).

#### Death cell counting

Trypan blue staining is used to identify dead cells which without intact membranes can be effectively stained. The 0.4% trypan blue solution was adopted to estimate the cell death rates of U118-R and T98G-R cells. The number of live (unstained) and dead (blue stained) cells can be analyzed using a hemocytometer on a basic upright microscope. The percent of cell death was calculated using the following equation: Cell death (%) = The number of dead cells/The total cell number × 100. Experiments were performed in triplicate and repeated three times with similar results. Bars display mean ± standard deviations (SD).

#### Clone forming testing

The U118-R and T98G-R cells were treated with ABCA1 siRNA for 24 h. Then, 1000 cells were counted and subsequently transferred into the six-well plates and made them clone for approximately 15 days with TMZ treatment at 100 μM. Finally, the clones were stained and counted with 0.3% crystal violet.

Meanwhile, 1000 cells were, respectively, counted and then seeded into 96-well plates for incubation (added with TMZ at 50 μM, 100 μM, 150 μM, 200 μM or 250 μM). After 72 h, Cell Counting Kit-8 (CCK-8) solution (10 μL; Beyotime, Jiangsu, China) was added to each well for 2 h. Subsequently, the optical density (OD) level at 450 nm was recorded with microplate reader.

#### Molecular docking

Molecular interactions between TMZ and ABCA1 were assessed using the Molecular Operating Environment (MOE) software (version 2019) to predict the affinity of ABCA1 and TMZ. Human serum albumin (HSA) has been reported by Rubio-Camacho et al. as a biomimetic carrier for TMZ due to its moderate interaction with TMZ and enhancement to the hydrolytic stability of TMZ [35]. Accordingly, the binding affinity between TMZ and HSA was utilized as the positive control to calculate the interaction strength of ABCA1 and TMZ. Data on TMZ 3D structure was downloaded from the PubChem database [36]. 3D structures of ABCA1 and human serum albumin were obtained from the RCSB PBD database [37]. The smallest negative number of S indicates the highest binding affinity.

### Correlation and enrichment analysis

Gene correlation and enrichment analysis were performed to understand the role of ABCA1 and the biological processes in glioma. The LinkedOmics database [38] integrates, analyzes, and compares the multi-omics data and clinical information across all TCGA tumors for genomics researchers. We conducted a gene correlation analysis associated with ABCA1 through the LinkFinder analytical module. Volcano plots and heat maps were employed to display the co-expressed genes of ABCA1. We simultaneously analyzed survival heat maps of the top 50 genes associated with ABCA1 via the GEPIA2 database. Moreover, another analytical module, the LinkInterpreter, carried out the gene set enrichment analysis, including gene ontology cellular component (GO-CC), gene ontology biological process (GO-BP), gene ontology molecular function (GO-MF) and Kyoto encyclopedia of genes and Genomes (KEGG) pathway.

### Immunological analysis

The Pearson correlation between ABCA1 expression and the abundance of immune cells in glioma tissues from the TCGA database was evaluated using the Xiantao tool, which applied the ssGSEA algorithm to estimate immune infiltration [39, 40]. Subsequently, the TISIDB [41] and HPA databases were utilized to verify the above immunological results. The Tumor Immune Single-cell Hub2 database (TISCH2) [42], providing a platform for investigating the tumor microenvironment, was also applied to further validate at the single-cell level. Moreover, we investigated the immune molecules correlated to ABCA1 expression using the Xiantao tool and TISIDB database. Additionally, TIMER2.0 [43], an integrated web database aiming to estimate the immune infiltration across various TCGA tumors systematically, was adopted to ascertain whether ABCA1 expression and immune infiltrates correlated with the clinical outcomes of glioma patients.

### Statistical analysis

GraphPad Prism (version 8.0.2) was used for statistical analysis. Kaplan–Meier analysis was applied to assess survival rate or probability for glioma patients. The unpaired *T*-test was adopted for the statistical hypothesis test to compare the differential expression level of ABCA1 in the glioma tissues and normal brain tissues. Correlation analysis included Pearson correlation test and Spearman correlation test. All experiments were conducted no less than three times with mean ± standard deviations (SD) for analysis. The *p*-value less than 0.05 was statistically significant (*p < 0.05, **p < 0.01, ***p < 0.001, and ****p < 0.0001).

## Results

### ABCA1 was identified as a co-differentially expressed gene from GEO datasets of glioma

**Fig. 1:**
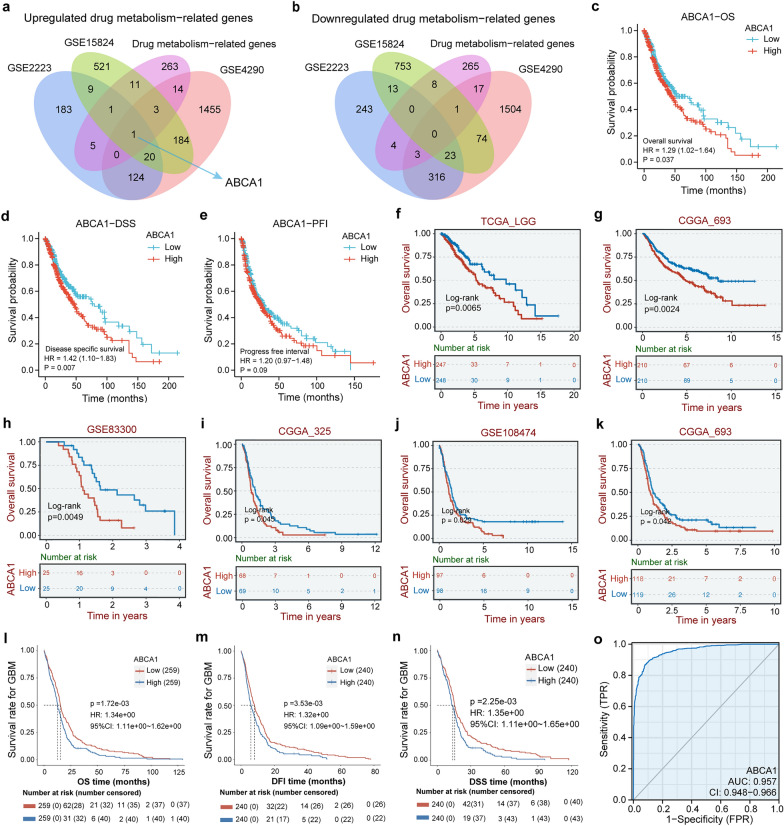
One co-differentially expressed gene (co-DEG) was identified and showed an excellent ability to predict clinical diagnosis and prognosis for glioma. **a**, **b** The number in every overlapping area represents the amount of differentially expressed genes. Only upregulated co-DEG, ABCA1, was identified from these datasets. **c-e** The survival probability of glioma in the TCGA database, including OS, DSS, and PFI, were respectively assessed. **f-k** Overall survivals of LGG (**f-g**) and GBM (**h–k**) in the TCGA, CGGA, and GEO databases were also obtained from the BEST database. **l-n** The survival plot for GBM in the TCGA database, including OS, DSS, and DFI, was attained from the PanCanSurvPlot tool. **o** The ROC curve implies that ABCA1 owns the diagnostic performance in discerning glioma tissue from normal tissue. Y-axis represents the true positive rate, and X-axis denotes the false positive rate. The numerical value of AUC closer to 1 indicates better diagnostic performance, significantly when exceeding 0.9

To ferret out the potential drug metabolism-related genes in glioma, we retrieved and analyzed three gene expression profiles of glioma and normal brain samples through the GEO database to acquire the differential expression genes with the screening criteria set as |logFC|> 1 and *p* < 0.01. As shown in Table 1, GSE15824 totally contains 30 glioma samples and five normal brain samples, in which 872 down-regulated and 750 up-regulated genes were screened out. GSE4290 embodies 157 glioma samples and 23 normal brain samples, with 1938 down-regulated and 1801 up-regulated genes screened out. GSE2223 includes 50 glioma and four normal brain samples, with 602 down-regulated and 343 up-regulated genes screened out. In the meantime, we collected certain drug metabolism-related genes reported to be involved in drug disposition, metabolism, and resistance to cancer chemotherapeutic agents [44]. Then, we conducted a Venn analysis to identify the co-DEGs from the above differentially expressed genes and the drug metabolism-related genes. One co-differentially expressed gene, ABCA1, was finally discovered and pended for further analysis (Fig. 1a, b).

### ABCA1 shows an excellent ability to predict clinical diagnosis and prognosis for glioma

We explored the ABCA1 associated clinical outcomes for glioma patients through several web databases and bioinformatics tools. We started with using the ABCA1 gene expression data and corresponding clinical information to assess the overall survival (OS), disease-specific survival (DSS), and progression-free interval (PFI) of glioma patients in the TCGA database. The result displays that glioma patients with higher ABCA1 expression levels have encountered worse OS, DSS, and PFI (Fig. 1c–e). Further investigation into OS, DSS, and disease-free interval (DFI) through the PanCanSurvPlot tool demonstrates the same result (Fig. 1l–n). To be more convincing, the BEST database was employed to examine the survival of glioma patients in the TCGA, GEO, and CGGA databases. The survival plots reveal lower expression levels of ABCA1 associated with better OS for glioma patients (Fig. 1f–k). Subsequently, we evaluated the diagnostic performance of ABCA1 in distinguishing glioma tissues from normal brain tissues through the ROC curve. The numerical value of AUC (0.957, 95% CI 0.861–0.923) indicates that ABCA1 has a superb capability in glioma diagnosis (Fig. 1o). These results initially imply that ABCA1 has important clinical significance for glioma patients and is worth profound investigation.

### ABCA1 expression level in glioma is more dramatically upregulated than in normal tissues

**Fig. 2:**
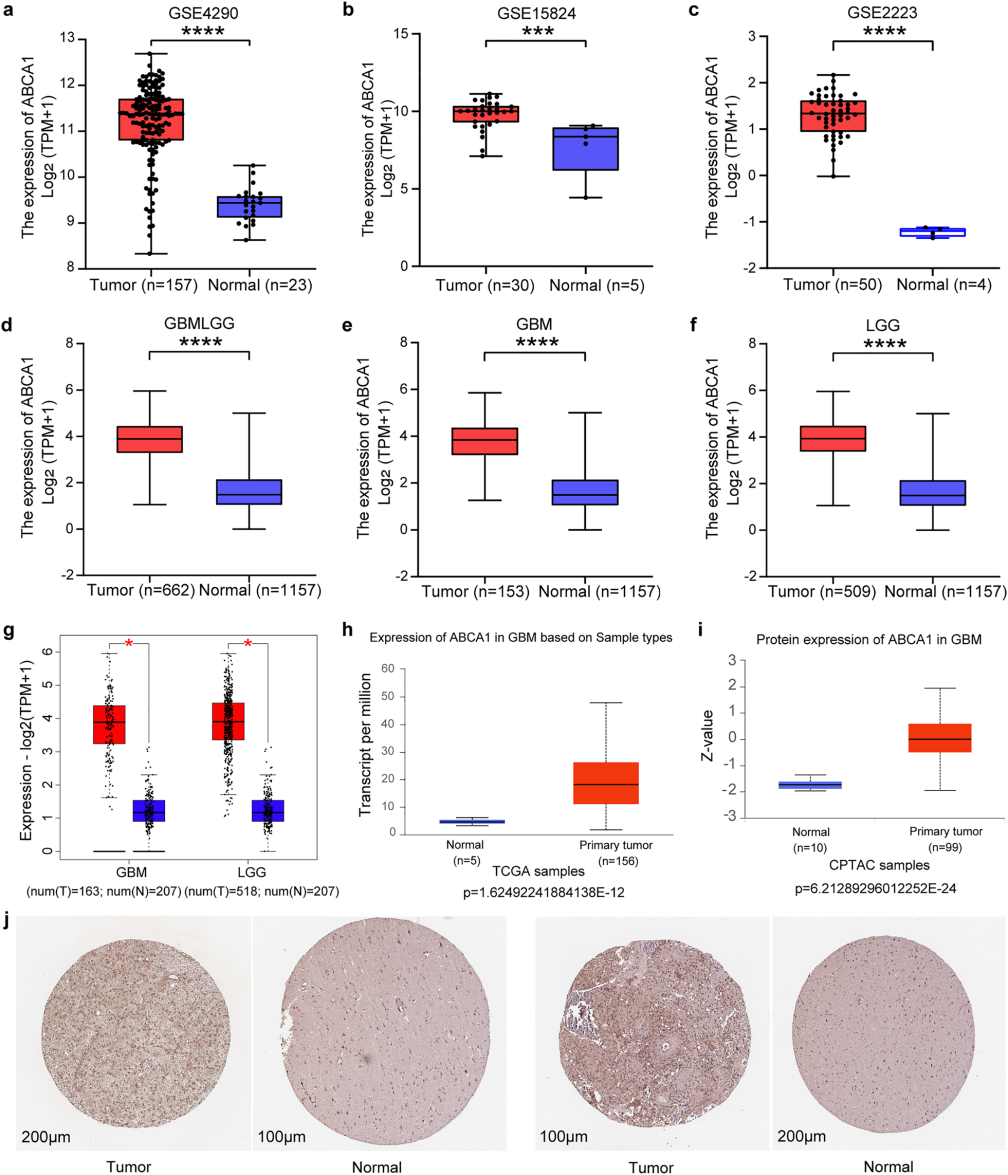
The increased expression levels of ABCA1 in glioma. **a-c** ABCA1 expression levels in glioma and normal brain samples from three GEO datasets. **d-f** ABCA1 expression levels in glioma and normal samples from TCGA and GTEx databases. **g** The plot from GEPIA 2 database displayed elevated ABCA1 expression levels in both GBM and LGG tissues compared to adjacent normal tissues. **h-i** The diagram from the UALCAN database showed upregulated transcript and protein expression levels in GBM samples. **j** Two group immunohistochemical images of ABCA1 from the HPA database also indicated an increased protein expression in tumor tissues. *p < 0.05, ***p < 0.001, ****p < 0.0001

According to the analysis of ABCA1 expression data in three GEO datasets and the TCGA database, we discovered that the expression level of ABCA1 in glioma was significantly elevated in comparison to the normal brain tissues (Fig. 2a–f). The analysis results based on 681 glioma tissues and 207 normal tissues from the GEPIA2 database display higher ABCA1 expression levels in glioma than in normal tissues (Fig. 2g). Additionally, data from the UALCAN database indicate that ABCA1 is over-expressed in glioma samples at the level of mRNA and protein (Fig. 2h, i). The immunohistochemical staining of ABCA1 shows intense staining in glioma tissues and weak in normal tissues, which reveals the highly expressed level of ABCA1 in glioma patients (Fig. 2j).

### ABCA1 has strikingly enhanced TMZ resistance in glioma

**Fig. 3:**
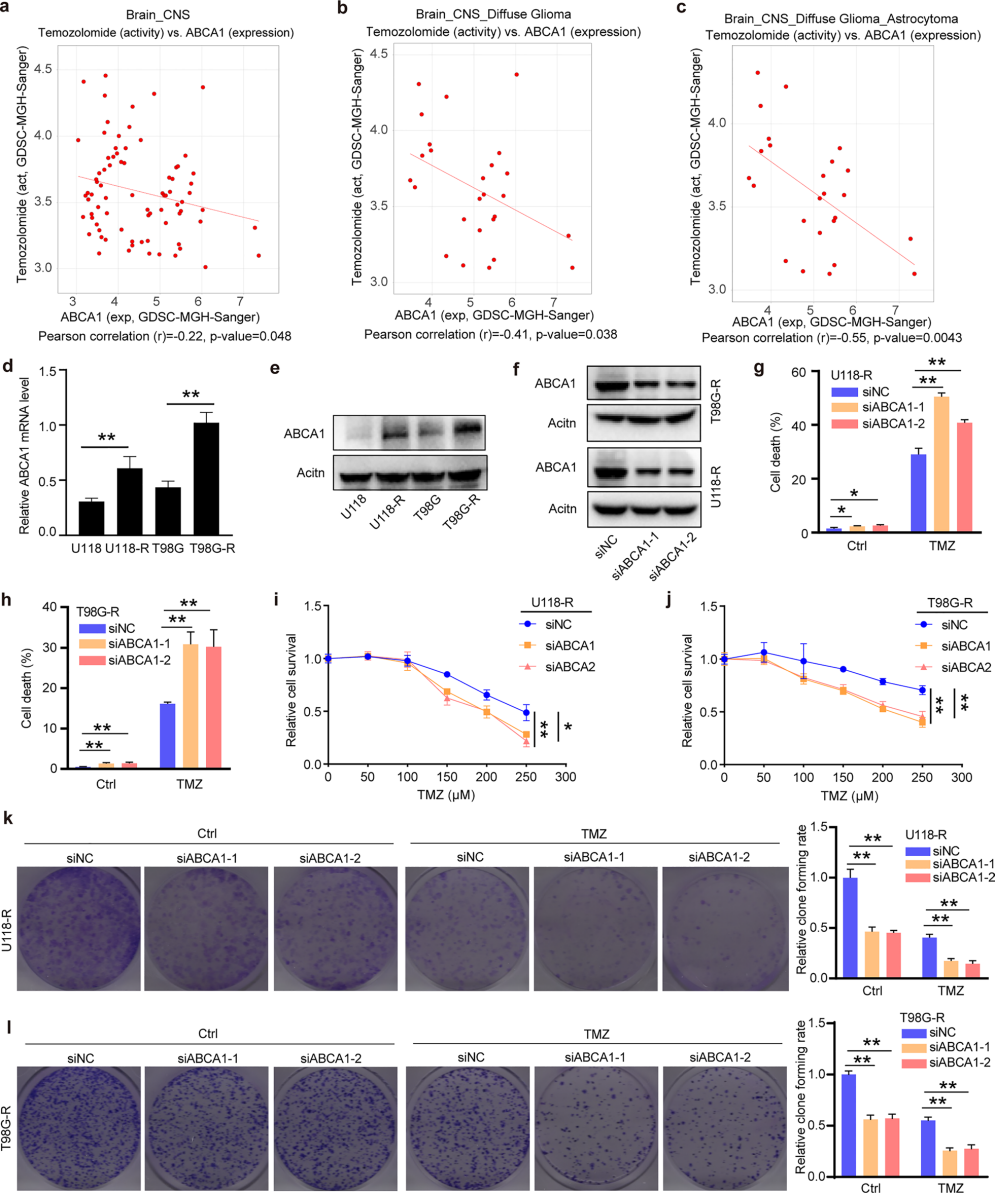
ABCA1 has strikingly enhanced TMZ resistance in glioma. **a–c** The diagram conducted by CellMinerCDB presents the negative correlation between ABCA1 expression level and the activity of TMZ. **d**,** e** In contrast with non-resistant cells (U118 and T98G), ABCA1 was significantly overexpressed in transcript and protein level across the TMZ-resistant cells (U118-R and T98G-R). **f** The western blotting indicated that ABCA1 expression was prominently decreased in TMZ-resistant glioma cells after knocking down certain base sequences GCTAGAACCCATAGCAACA (siABCA1-1) and CCAGCCAGCTTTGTCGTAT (siABCA1-2). **g**, **h** ABCA1 knockdown combined with TMZ treatment could facilitate the death rate of U118-R and T98G-R cells. **i**, **j** The relative cell survival of U118-R and T98G-R cells detected using CCK-8 significantly reduced with the higher treatment level of TMZ, and declined more sharply after ABCA1 knockdown. **k**, **l** ABCA1 knockdown and TMZ treatment at 100 μM restrained the clone-forming rate of U118-R and T98G-R cells. *p < 0.05, **p < 0.01

The CellMinerCDB platform conducted a correlativity analysis of ABCA1 expression and TMZ activity in glioma cell lines to investigate whether ABCA1 augmented TMZ resistance in the treatment of glioma patients. The diagram exhibits that ABCA1 expression remarkably negatively associates with TMZ resistance in central nervous system tumors, especially in diffuse glioma (Pearson *r* = − 0.41, *p* = 0.038) and astrocytoma (Pearson *r* = − 0.55, *p* = 0.0043) (Fig. 3a–c). Cell experiments were followed for further exploration and verification of the above analysis. The mRNA expression data measured by real-time PCR revealed that the expression level of ABCA1 in TMZ-resistant glioma cells (U118-R and T98G-R) was significantly higher than that in non-resistant glioma cells (U118 and T98G) (Fig. 3d). The results of analyzing the protein expression levels of ABCA1 with Western blotting showed that ABCA1 in TMZ-resistant glioma cells was significantly higher than that in non-resistant glioma cells (Fig. 3e). Reduced expression levels of ABCA1 (siABCA1-1 and siABCA1-2) in TMZ-resistant glioma cells U118-R and T98G-R were detected by Western blotting, which indicated that ABCA1 expression successfully suffered from interference and knockdown (Fig. 3f). Cell death of U118-R and T98G-R cells increased after ABCA1 expression had been interfered by siRNA. After ABCA1 got knocked down, treatment with TMZ at 100 μM could prominently facilitate the death rate of U118-R and T98G-R cells (Fig. 3g, h). The relative cell survival of U118-R and T98G-R cells significantly reduced with the treatment higher level of TMZ, and declined more sharply after ABCA1 knockdown (Fig. 3i, j). Moreover, the cell clone forming rate of U118-R and T98G-R cells decreased after ABCA1 knockdown. Based on ABCA1 knockdown, treatment with TMZ at 100 μM strikingly inhibited the clone forming of U118-R and T98G-R cells (Fig. 3k, l). The data of colony formation analysis and CCK-8 showed ABCA1 knockdown significantly reduced proliferation and colony formation ability of glioma cells.

**Fig. 4:**
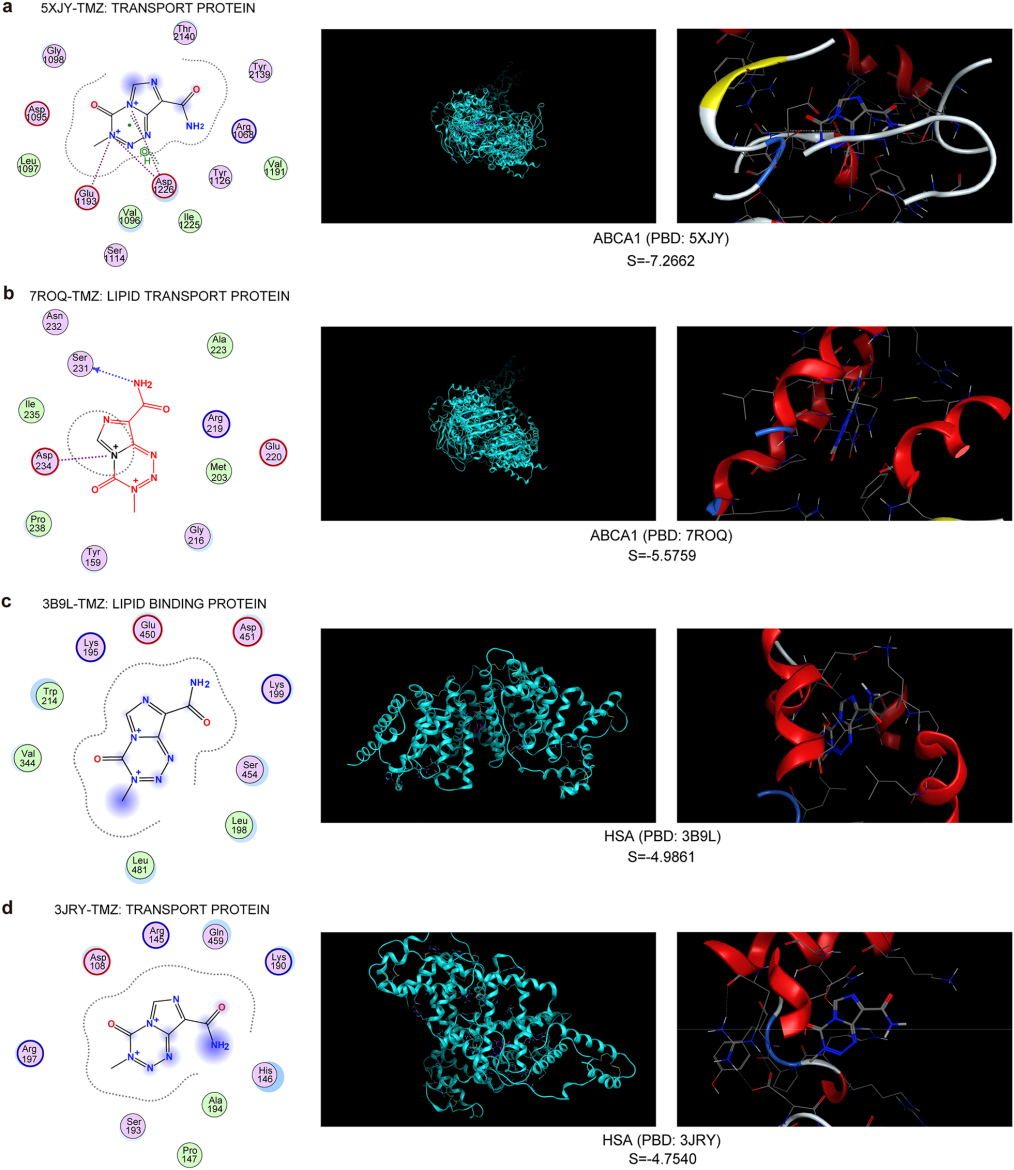
Calculation of TMZ binding to different protein conformations of ABCA1 and human serum albumin (HSA) using MOE software (version 2019). **a** TMZ binds to PBD_5XJY of ABCA1. **b** TMZ binds to PBD_7ROQ of ABCA1. **c** TMZ binds to PBD_3B9L of HSA. **d** TMZ binds to PBD_3JRY of HSA

Subsequently, MOE software was employed to predict the affinity of ABCA1 and TMZ. HSA was selected as a positive control to assess the binding intensity of TMZ to ABCA1. The negative number of S was negatively correlated to the binding affinity between TMZ and proteins. The results of molecular docking between TMZ and different protein structures of ABCA1 displayed that the smallest negative numbers of S were − 7.2662 (PBD_5XJY, Fig. 4a) and − 5.5759 (PBD_7ROQ, Fig. 4b). As to HSA in different protein conformations, the smallest negative numbers of S were − 4.9861 (PBD_3B9L, Fig. 4c) and − 4.7540 (PBD_3JRY, Fig. 4d). Other docking results are shown in Table 2. The docking scores of ABCA1 and TMZ were relatively high compared to HSA and TMZ. These results indicated that the interaction strength of TMZ and ABCA1 is greater than that of TMZ and HSA.

**Table 2:** Molecular docking results of temozolomide with ABCA1 and human serum albumin (HSA)

PDB	Molecule	S	Rmsd_refine	E_confo	E_place	E_score1	E_refine
ABCA1 _5XJY		− 7.2662	1.1088	− 254.9061	− 48.9719	− 10.6095	60.8538
ABCA1 _7ROQ		− 5.5759	1.4952	− 263.7835	− 62.8099	− 8.5023	82.9998
ABCA1 _7TBY		− 5.0127	1.2826	− 263.6897	− 66.9917	− 8.3575	− 0.6379
HAS _3B9L		− 4.9861	1.1354	− 262.1237	− 61.6598	− 10.6449	− 5.7354
HAS _3JRY		− 4.7540	2.3088	− 255.9423	− 54.4165	− 8.8933	− 16.5218
HAS _6EZQ		− 4.5677	0.6975	− 254.7766	− 67.7218	− 9.9736	− 1.5844

### Co-expression genes and biological functions of ABCA1 in glioma

**Fig. 5:**
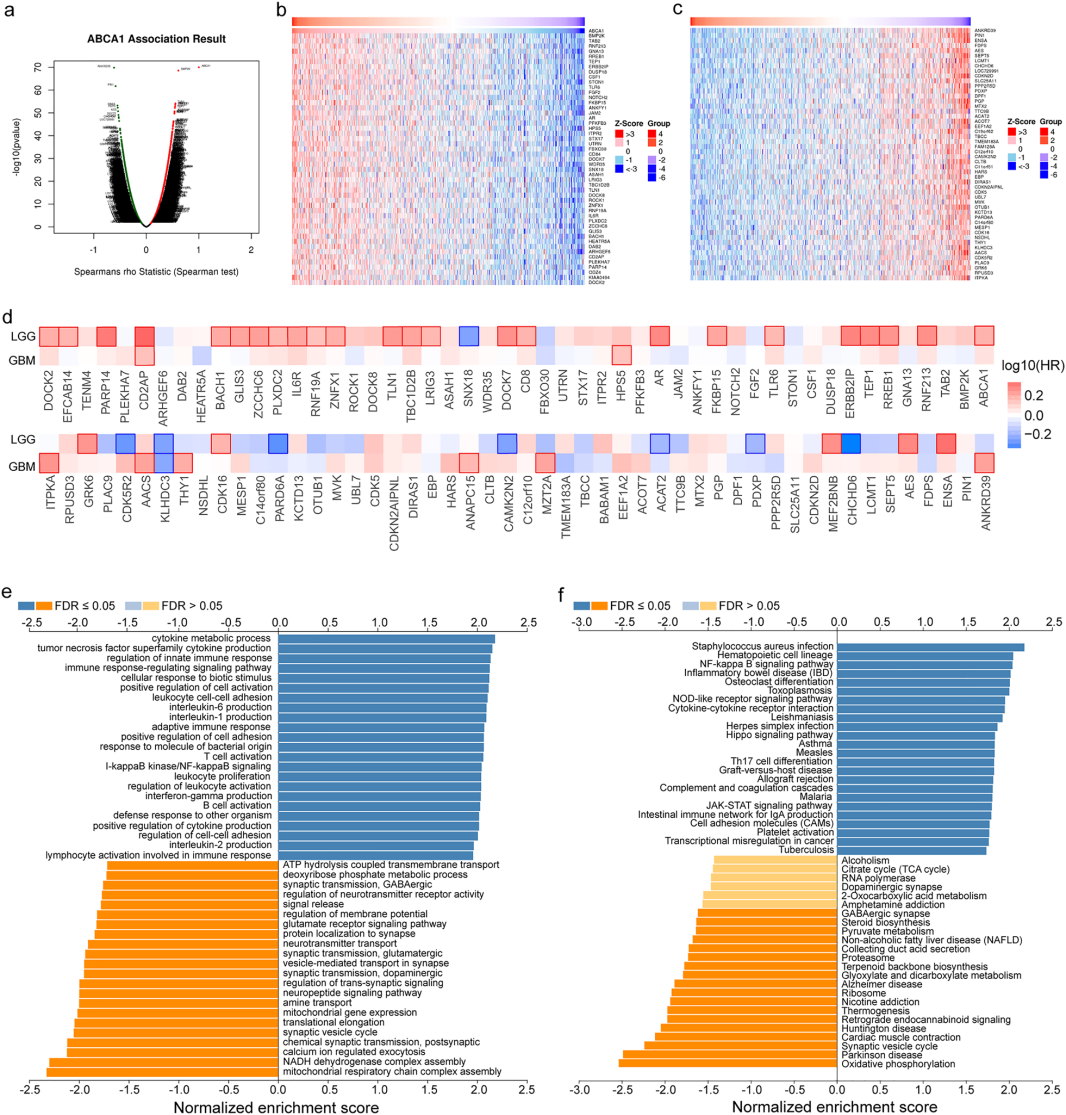
ABCA1-associated co-expressed genes and enrichment analysis in glioma. **a** The whole genes significantly associated with ABCA1 in glioma were identified by the LinkedOmics database. **b**, **c** Heatmaps showed the top 50 genes positively and negatively associated with ABCA1 in glioma. The red color indicates positively associated genes, whereas the blue shows negatively. **d** Survival heat maps presented the top 50 genes positively and negatively associated with ABCA1 in glioma. **e**, **f** GO annotations of biological process and KEGG pathways correlated with ABCA1

The LinkedOmics database was adopted for gene correlation and enrichment analysis of the TCGA glioma to investigate the biological roles of ABCA1 in glioma. Figure 5a shows that 6785 genes were positively associated with ABCA1, while 6381 genes were negatively correlated with ABCA1 (*p* < 0.05). Additionally, the heatmaps presented the top 50 genes associated with ABCA1 negatively and positively (Fig. 5b, c). The results of survival heatmaps showed that positive co-expressed genes with a risky hazard ratio (25/50) acted as risk factors. In contrast, negative co-expressed genes with a protective ratio (7/50) were more likely to be protective factors (Fig. 5d). GO annotations of the biological process described that ABCA1 was chiefly involved in the process of immune regulation and mitochondrial metabolism, such as cytokine metabolism, cytokine production, immune cell activation, complex assembly, and so on (Fig. 5e). Results of KEGG pathways depicted the top pathways that ABCA1 participated in, including oxidative phosphorylation, Parkinson’s disease, and infection (Fig. 5f). Moreover, GO annotations of cellular components and molecular function indicated that ABCA1 mainly functioned in immune regulation and mitochondrial metabolism (Additional file 1: Fig. S1a, b).

### Overexpressed ABCA1 stimulates the infiltration of macrophages (M2) in glioma.

**Fig. 6:**
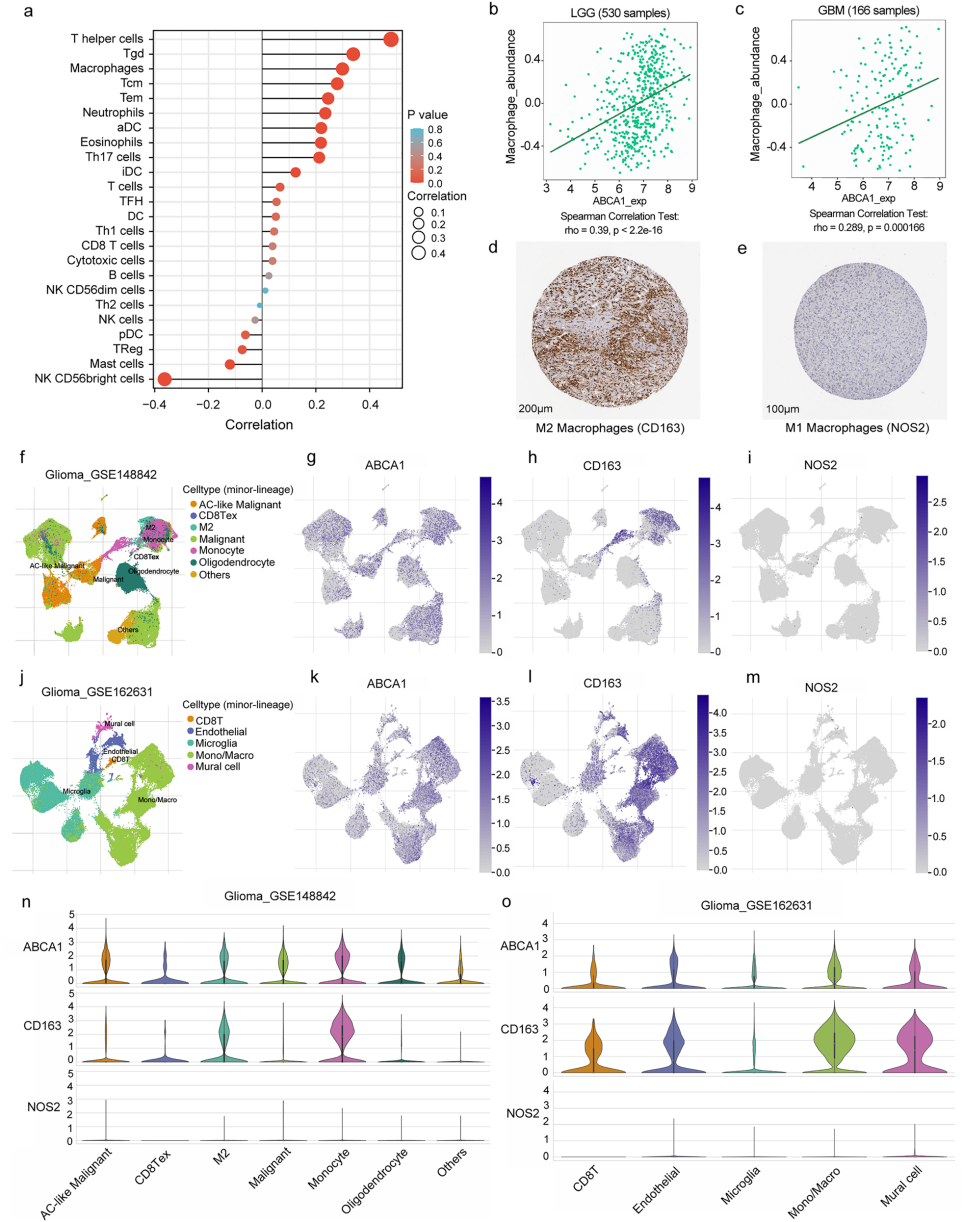
Correlation of ABCA1 expression with immune cell infiltration in glioma. **a** Association between ABCA1 and various tumor immune-infiltrating cells in TCGA database. **b**, **c** Association between ABCA1 and the abundance of macrophages. **d**, **e** Based on the immunohistochemical staining of specific cell markers from the HPA database, M2 macrophages (CD163) were intensively detected in glioma tissues, while M1 macrophages (NOS2) were not nearly detected. **f–o** The diagram drawn by the TISCH2 platform displays the cell types and proportions in glioma tissues at the single-cell level. Monocytes/macrophages (mainly M2 macrophages, CD163) are the preponderant immune cells infiltrating glioma tissues

A chart displayed the correlation analysis results of ABCA1 and immune cell infiltration. It revealed that ABCA1 expression had a positive correlation with the abundance of T helper cells, T gamma delta, macrophages, T effector memory cells, T central memory cells, neutrophils, activated dendritic cells (aDC), eosinophils and Th17 cells in glioma (*p* < 0.05) (Fig. 6a). Then, the above results were verified by the TISIDB database. The consistent correlation of ABCA1 expression was discovered with the infiltration level of macrophages, neutrophils, aDC, and Th17 cells (Fig. 6b, c**, **Additional file 2: Fig. S2a–f). Notably, a difference existed in that CD163 (CD163 molecule; M2 macrophages), HLA-DPB1 (major histocompatibility complex, class II, DP beta 1; aDC), and STAT (signal transducer and activator of transcription; Th17 cell) were intensely stained in the immunohistochemistry images of glioma tissues, while NOS2 (nitric oxide synthase 2; M1 macrophages) and CD66b (CEACAM8, CEA cell adhesion molecule 8; neutrophil) were nearly not detected (Fig. 6d, e**, **Additional file 2: Fig. S2g–i). Hence, we used the TISCH2 database to intensively investigate the cell types of immune infiltration in glioma samples from the datasets of GSE148842 and GSE162631 at the single-cell level. The diagram depicted that across the immune cells infiltrating the glioma tissues, the preponderant ones were monocytes/macrophages (mainly M2 macrophages), and other immune cells (CD8 T cells and M1 macrophages) were few or even undetectable (Fig. 6f–o). All the results revealed that a mounting infiltration abundance of M2 macrophages in glioma was primarily related to the highly expressed level of ABCA1.

**Fig. 7:**
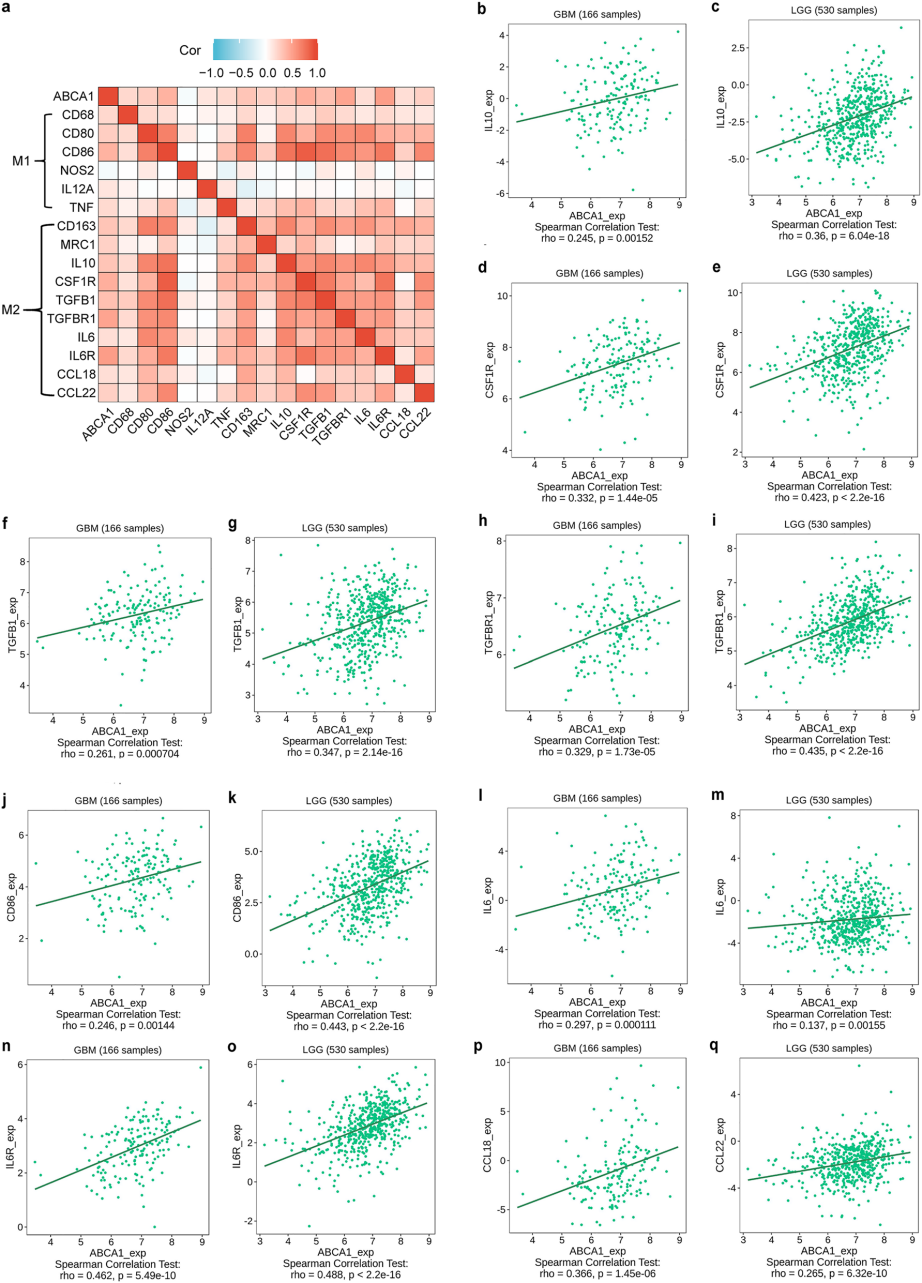
Relationship of ABCA1 with immune molecules related to M2 macrophages in glioma. **a** Correlation between ABCA1 and immune molecules associated with M2 macrophages. **b-q** Expression correlation between ABCA1 and the immunoinhibitors (IL-10, CSF1R, TGFB1, TGFBR1), immunostimulators (CD86, IL-6, IL-6R), and chemokines (CCL18, CCL22)

Based on the notable correlation of ABCA1 with immune infiltration, we explored the association between ABCA1 and immune molecules related to macrophages in GBM and LGG [45]. Immunoinhibitors (IL-10, interleukin 10; CSF1R, colony stimulating factor 1 receptor; TGFB1, transforming growth factor beta 1; TGFBR1, transforming growth factor beta receptor 1), immunostimulators (CD86, CD86 molecule; IL-6, interleukin 6; IL-6R, interleukin 6 receptor), and chemokines (CCL18, C–C motif chemokine ligand 18; CCL22, C–C motif chemokine ligand 22) own positive correlations with ABCA1 expression (Fig. 7). Additionally, we explored whether immune infiltrates owned a potential correlation with the clinical outcomes of glioma patients. The results displayed that at a certain level of gene expression, macrophages with high infiltrating abundance acted as an adverse hazard ratio and were associated with poor cumulative survival in patients with glioma (Additional file 2: Fig. S2j, k).

## Discussion

Gliomas are a class of aggressive primary intracranial tumors, presenting high morbidity with a prevalence of 42.8% in central nervous system tumors and alarming mortality with a 5-year relative survival rate lower than 35.7% in HGG [1, 46]. The management of glioma has currently advanced to multidisciplinary involvement, including resection of tumors, radiotherapy, chemotherapy, and immunotherapy. Chemotherapy with TMZ plus radiotherapy or surgery has been proven effective in extending the survival time and improving the quality of life in glioma patients [47, 48]. The main challenge is that most cases have encountered treatment limitations or failure due to the subsequent emergence of low chemosensitivity, known as drug resistance. Nevertheless, TMZ remains the first-choice alkylating drug in standard regimens used alone or concomitant to other treatments because of its superior ability to penetrate the blood–brain barrier [49]. Hence, enunciating the resistant mechanism is imperative to overcome the unsatisfied chemosensitivity and enhance the therapeutic efficacy of TMZ in glioma treatment.

The potential mechanisms have been reported in extensive studies as follows. High metabolism demands and hypoxic environment of glioma stimulate the emergence of angiogenic mediators and abnormal angiogenesis in the blood–brain tumor barrier. Dysfunctional new blood vessels promote the transfer of nutrition and oxygen while obstructing chemotherapeutic agents from the blood to tumors, thus contributing to tumor growth and expansion [50]. Activation of the DNA repair system induced by TMZ maintains the cell genome of gliomas integrated and stable, such as DNA repair mediated by O^6^-methylguanine DNA methyltransferase, DNA mismatch repair performed by the repair complex, and DNA base excision repair conducted by repair proteins [51–53]. The existence of glioma stem cells with characteristics related to self-renewal, differentiation, metastasis, invasion, DNA repair, and resistance to chemoradiotherapy contributes to tumor proliferation or progression [32]. Activated self-protective autophagy of glioma cells triggers chemoresistance by decreasing the cytotoxicity and antitumor effect of TMZ [54, 55]. Selective pressure on exposure to chemotherapy agents enhances the expression level of drug efflux transporters, resulting in unsatisfactory chemosensitivity [32]. To summarize, previous research on the mechanism of TMZ resistance involves the dysfunctional blood–brain barrier, DNA repair system, glioma stem cells, self-protective autophagy, drug efflux transporters, etc.

Our research centered on screening drug metabolism-related proteins from several bioinformatics datasets and elucidating their role in the TMZ resistance of glioma treatment through multi-omics analysis and experiments. An upregulated drug metabolism-related protein, efflux transporter protein ABCA1, was discovered in glioma datasets. The significantly over-expressed mRNA and protein levels of ABCA1 exist in glioma tissues compared to normal brain tissues. Among the patients with glioma, the survival prognosis in high-expression groups of ABCA1 is prominently inferior to that in low-expression groups. Meanwhile, ROC analysis indicates that ABCA1 shows an exceptional diagnostic value and performance in distinguishing glioma tissues from normal brain tissues with the numerical value of AUC up to 0.957 (95% CI 0.861–0.923). Moreover, existing studies have reported that overexpressed ABCA1 facilitates tumor cell proliferation, tumor growth, and acquired chemotherapy resistance in multiple tumors [13–15, 18]. Yet treatment with inhibitor apabetalone ameliorates cell proliferation, tumor progression, and chemotherapy resistance. Accordingly, ABCA1 may also promote chemoresistance of glioma through the mechanism of efflux of TMZ, and its role is worthy of further investigation.

According to the mentioned findings, we conducted a correlation analysis between ABCA1 expression and the activity of TMZ in glioma cell lines. The result demonstrates that the higher the level of ABCA1 expressed in glioma cells, the lower the activity of TMZ. Besides, we detected relatively higher mRNA and protein levels of ABCA1 in TMZ-resistant glioma cells (U118-R and T98G-R) compared to the non-resistant glioma cells (U118 and T98G). The protein levels of ABCA1 were notably reduced in U118-R and T98G-R cells after ABCA1 knockdown. The relative clone forming rate and cell survival of U118-R and T98G-R cells was also impaired, and the death rate rose accordingly. In addition, the above effects were more manifest when TMZ was administered to the U118-R and T98G-R cells with ABCA1 knockdown. This means that the chemoresistance of TMZ was ameliorated when ABCA1 gets knockdown or inhibited. Moreover, the results of molecular docking revealed a relatively high affinity between TMZ and ABCA1 transport protein. These results indicate that ABCA1 restrains the chemosensitivity of TMZ in glioma cells. Hence, concomitant treatment with TMZ and ABCA1 inhibitors may be feasible to tackle chemoresistance and enhance the chemotherapy effect.

The following is to investigate the role of ABCA1 transporter in glioma through co-expression and pathways analysis. Half of the positively associated co-expression genes are risk factors, whereas a small proportion of the negative ones acts as protective factors. The enrichment analysis indicates that ABCA1 is mainly involved in immune regulation, such as cytokine metabolic process, cytokine production, cytokine binding, cytokine receptor activity, innate immune response, immune signaling pathway, and infection. We thereupon explored the correlation between ABCA1 and tumor-immune infiltration cells in glioma from multiple levels. Based on the markers of immune cells, we finally found an abundance of M2 macrophages infiltrating in glioma tissues with high levels of ABCA1. A high infiltrating abundance of macrophages predicts an adverse hazard ratio and poor survival in patients with LGG and GBM. And immune molecules related to macrophages positively correlate with ABCA1, such as IL-10, TGFB1, TGFBR1, CD86, IL-6, IL-6R, etc.

Macrophages have been reported to promote tumorigenesis and predict poor clinical outcomes in glioma patients [56, 57]. It has been studied that macrophages exhibiting high versatility will polarize under different stimuli into two subsets: pro-inflammatory M1 and anti-inflammatory M2 macrophages [58]. M1 macrophages express the surface markers of CD68 (CD68 molecule), CD86, CD80 (CD80 molecule), and NOS2, secrete the cytokines such as TNF (tumor necrosis factor) and IL-1β (interleukin 1 beta), and mediate antitumor resistance and tissue damage. In constant, M2 macrophages express the surface markers of CD163, CD86, and CD206 (MRC1, mannose receptor C-type 1), secrete cytokines including IL-10, TGF-β, and IL-6, and function in wound repair and tumor growth [45, 59]. Among all the immune molecules involved, IL-10 has been intensively studied as a biomarker of M2 macrophages [58]. Therefore, the result that ABCA1 is positively associated with IL-10 confirms the positive correlation of ABCA1 with M2 macrophage infiltration in glioma. To sum up, ABCA1 may facilitate the immune infiltration of M2 macrophages in glioma by inducing specific cytokines production and metabolism, thereby supporting tumor escape and growth and causing unsatisfactory survival outcomes for patients. However, further in vitro and in vivo experiments are required to investigate and confirm this finding.

## Conclusions

We concentrated on identifying the drug metabolism-related genes involved in the in vivo process of TMZ and elucidating their roles in the chemoresistance of glioma treatment. Our study discovered an overexpressed level of ABCA1 in the glioma tissues and TMZ-resistant cells deriving from glioma. High expression levels of ABCA1 indicate poor prognosis in glioma patients and unsatisfied chemosensitivity of TMZ. ABCA1 knockdown followed by treatment with TMZ suppressed the growth of resistant cells and improved the chemotherapy efficacy. Moreover, ABCA1 in glioma may facilitate the immune infiltration of M2 macrophages which promote tumorigenesis and predict poor clinical outcomes for patients. Our findings indicate that ABCA1 knockdown or inhibition is a potential strategy for improving the therapeutic efficacy of gliomas.

### Supplementary Information

**Additional file 1: Figure S1.** Co-expression analysis of ABCA1 in glioma. (**a**) GO annotations of cellular component. (**b**) GO annotations of molecular function.

**Additional file 2: Figure S2.** Correlation between ABCA1 expression and the abundance of lymphocytes and clinical outcomes related to macrophage infiltrating in glioma. **a**–**f** Association between ABCA1 and the abundance of lymphocytes, namely neutrophils, Th17 cells, and active dendritic cells. **g**–**i** Based on the immunohistochemical staining of specific cell markers from the HPA database, Th17 cells (STAT) and dendritic cells (HLA-DPB1) were respectively detected in glioma tissues, while neutrophils (CD66b) were not detected. **j**–**k** Cumulative survival associated with ABCA1 expression and immune cell infiltration in glioma was assessed using TIMER 2.0.

**Additional file 3: Table S1.** Bioinformatics technologies used in this study.

## Data Availability

The datasets used and analyzed during the current study are available from the corresponding author on reasonable request.

## Abbreviations

TMZ: Temozolomide
GBM: Glioblastoma multiforme
GEO: Gene Expression Omnibus
AUC: The area under the curve
GO: Gene ontology
ROC: Receiver operation characteristic
DEGs: Differentially expressed genes
GEPIA2: Gene Expression Profiling Interactive Analysis 2
HPA: The Human Protein Atlas
BEST: Biomarker Exploration of Solid Tumors
TISCH2: Tumor Immune Single-cell Hub2
HGG: High-grade glioma
LGG: Low-grade glioma
GTEx: Genotype-Tissue Expression
TCGA: The Cancer Genome Atlas
HAS: Human serum albumin
ABCA1: ATP-binding cassette transporter subfamily A1
UCSC: University of California, Santa Cruz
UALCAN: University of Alabama at Birmingham Cancer
PBD: Protein Data Bank
MOE: Molecular Operating Environment
TISIDB: Tumor-Immune System Interactions Database
