# Supplementary figures and images for: Drug metabolism-related gene ABCA1 augments temozolomide chemoresistance and immune infiltration abundance of M2 macrophages in glioma
Source: Eur J Med Res. 2023 Sep 25;28:373. doi: 10.1186/s40001-023-01370-6 (PMC10518970; doi:10.1186/s40001-023-01370-6)

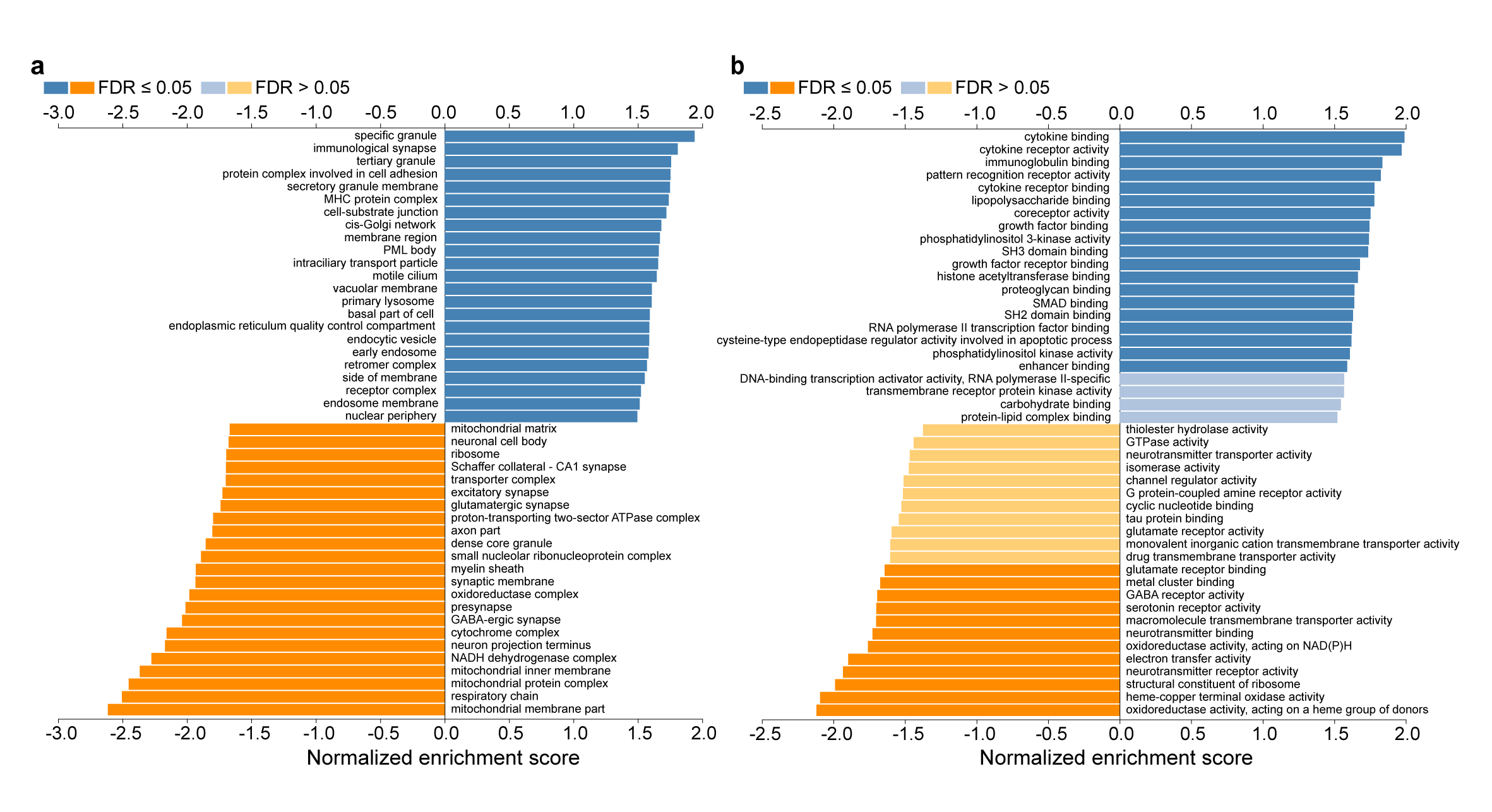

Supplement: Supplementary file 1 — Additional file 1: Figure S1. Co-expression analysis of ABCA1 in glioma. (a) GO annotations of cellular component. (b) GO annotations of molecular function. [file 40001_2023_1370_MOESM1_ESM.tif]

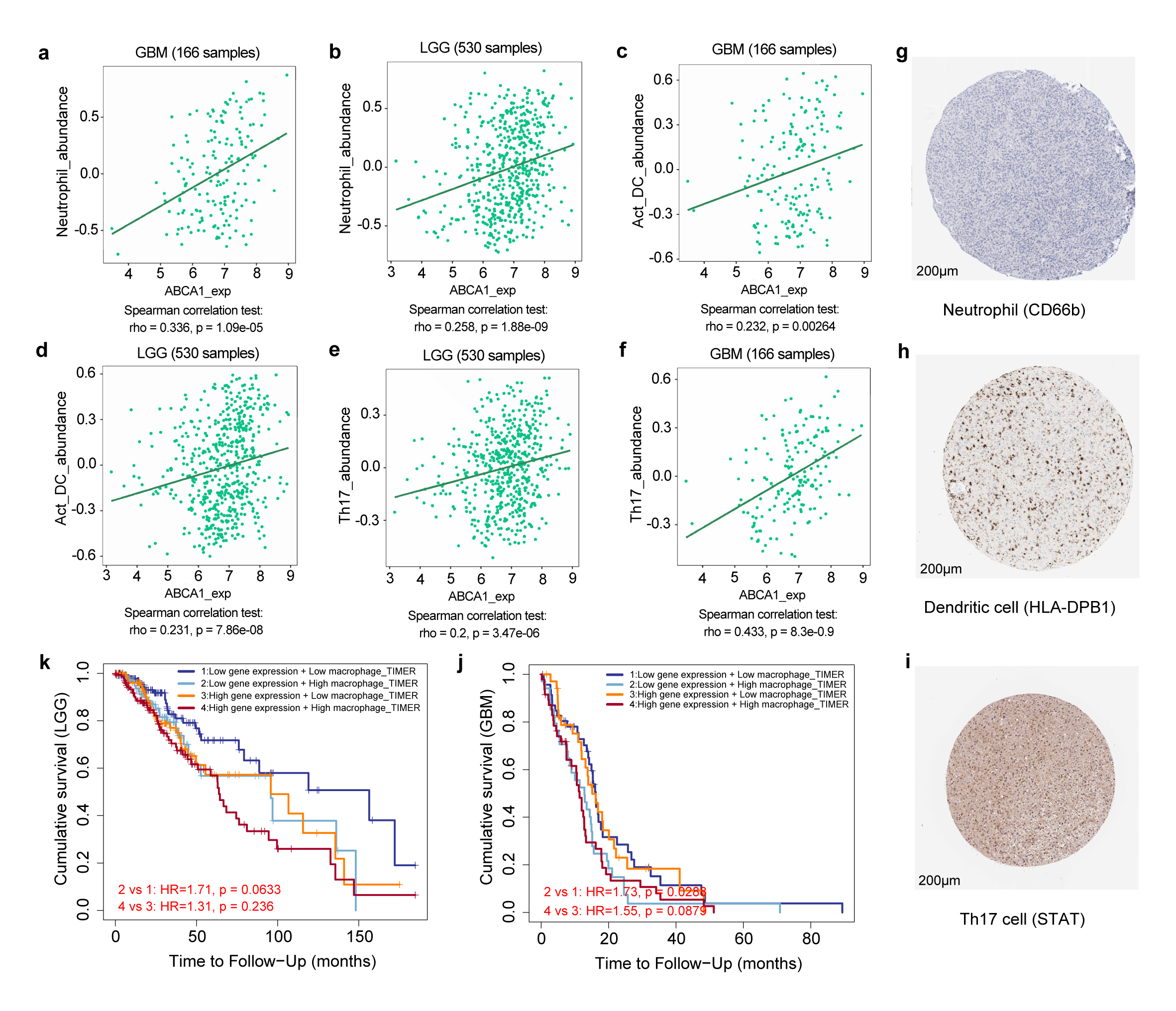

Supplement: Supplementary file 2 — Additional file 2: Figure S2. Correlation between ABCA1 expression and the abundance of lymphocytes and clinical outcomes related to macrophage infiltrating in glioma. a–f Association between ABCA1 and the abundance of lymphocytes, namely neutrophils, Th17 cells, and active dendritic cells. g–i Based on the immunohistochemical staining of specific cell markers from the HPA database, Th17 cells (STAT) and dendritic cells (HLA-DPB1) were respectively detected in glioma tissues, while neutrophils (CD66b) were not detected. j–k Cumulative survival associated with ABCA1 expression and immune cell infiltration in glioma was assessed using TIMER 2.0. [file 40001_2023_1370_MOESM2_ESM.tif]
