# Supplementary material for: Drug metabolism-related gene ABCA1 augments temozolomide chemoresistance and immune infiltration abundance of M2 macrophages in glioma
Source: Eur J Med Res. 2023 Sep 25;28:373. doi: 10.1186/s40001-023-01370-6 (PMC10518970; doi:10.1186/s40001-023-01370-6)
Supplement: Supplementary file 3 — Additional file 3: Table S1. Bioinformatics technologies used in this study. [file 40001_2023_1370_MOESM3_ESM.docx]

**Table S1.** Bioinformatics technologies used in this study.

| Operation process | Databases and tools | Links | Access date |
| --- | --- | --- | --- |
| Data acquisition | GEO | https://www.ncbi.nlm.nih.gov/geo/ | 07/10/2022 |
|  | UCSC | https://xenabrowser.net/ | 04/11/2022 |
|  | TCGA | https://portal.gdc.cancer.gov/ | 04/11/2022 |
| Venn analysis | Omicstudio | https://www.omicstudio.cn/tool/6 | 16/10/2022 |
| Survival analysis | Xiantao | https://www.xiantaozi.com/ | 16/02/2023 |
|  | BEST | https://rookieutopia.com/app_direct/BEST/ | 14/10/2022 |
|  | PanCanSurvPlot | https://smuonco.shinyapps.io/PanCanSurvPlot/ | 14/02/2022 |
| Expression analysis | GraphPad Prism 8.0.2 | https://www.graphpad.com/ |  |
|  | GEPIA2 | http://gepia2.cancer-pku.cn/#index | 06/10/2022 |
|  | UALCAN | http://ualcan.path.uab.edu/ | 06/10/2022 |
|  | HPA | https://www.proteinatlas.org/ | 01/02/2022 |
| TMZ activity analysis | CellMinerCDB 1.6 | https://discover.nci.nih.gov/rsconnect/cellminercdb/ | 11/02/2022 |
|  | RCSB Protein Data Bank | https://www.rcsb.org/ | 17/03/2023 |
| Enrichment analysis | LinkedOmics | http://www.linkedomics.org/login.php | 14/10/2022 |
| Immunological analysis | TISIDB | http://cis.hku.hk/TISIDB/index.php | 26/03/2023 |
|  | TISCH2 | http://tisch.comp-genomics.org/home/ | 20/02/2023 |
|  | TIMER 2.0 | http://timer.comp-genomics.org/ | 13/10/2022 |
